# Evaluation of psychological effects of orthodontic treatment vs. orthognathic surgery: a systematic review and meta-analysis

**DOI:** 10.1186/s12903-026-08040-z

**Published:** 2026-03-11

**Authors:** Niccolò Cenzato, Francesca Alicchio, Antonino Manti, Benedetta Baldini, Massimo Del Fabbro, Cinzia Maspero

**Affiliations:** 1 Department of Biomedical, Surgical and Dental Sciences, University of Milan, Milan, 20122 Italy; 2 Fondazione IRCCS Ca’ Granda, Ospedale Maggiore Policlinico, Milan, 20122 Italy; 3 Department of Electronics, Information and Bioengineering, Politecnico di Milano, Milan, 20133 Italy

**Keywords:** Orthodontic treatment, Orthognathic surgery, Psychological profile, Self-esteem, Orthodontic Patient expectations, Oral health-related quality of life (OHRQoL)

## Abstract

**Objective:**

This systematic review and meta-analysis aimed to evaluate the psychological impact of orthodontic treatment (OT) versus orthognathic surgery (OST).

**Materials and methods:**

A systematic review was conducted in accordance with PRISMA guidelines, using the PICOS framework to define the inclusion criteria for comparing the psychological impact of orthodontic treatment (OT) versus orthognathic surgery (OST). Searches were performed across PubMed, MEDLINE, EMBASE, PsychInfo, CINAHL, and CENTRAL databases for studies published between 2014 and 2025. A meta-analysis was performed on OT and OST studies reporting mean and standard deviation from the OHIP-14 questionnaire. A separate meta-analysis was performed for OST studies using OQLQ.

**Results:**

15 studies satisfied the predetermined inclusion criteria. OT was associated with improvements in OHRQoL over the course of treatment. These changes became more evident at later follow-up points. OST provided more immediate and pronounced OHRQoL improvements, particularly in psychosocial dimension such as self-esteem and social interactions. The OHIP-14 meta-analysis (ten studies) showed a mean OHRQoL improvement of 9.66 points, with high heterogeneity (I² = 97.91%) and no significant difference between OT and OST. The OQLQ meta-analysis (six studies) showed a 19.50 point improvement (I² = 96.80%) in psychological outcomes. Variability was attributed to differences in study design, tools, and patient characteristics.

**Conclusions:**

Both OST and OT improve OHRQoL, although through different mechanisms and timelines. OST offers faster psychological benefits. OT provides slower gains with less psychological discomfort, especially with aligners. Personalized pre-treatment education is essential and further research is needed to explore long-term psychosocial outcomes.

## Introduction

The growth and development of the orofacial region may deviate from ideal patterns due to genetic or environmental factors, potentially affecting oral functions and dental occlusion. Patients with malocclusion often report psychosocial concerns. These issues may arise from alterations in chewing, speech, swallowing and the overall facial and dental aesthetics.

Previous studies demonstrated a positive association between physical attractiveness and interpersonal relationships, social acceptance and self-perception, particularly in individuals with harmonious smiles and well-aligned teeth [1–3]. Conversely, malocclusion may have important social consequences and psychological effects, inducing low self-esteem and in some cases impairing patient’s socialization skills, worsening the quality of life (QoL) and psychosocial impact [1–3].

The management of dentofacial deformities varies according to patient age and severity: in growing patients, interceptive orthopaedic-orthodontic treatment is often indicated, whereas in adult patients a multidisciplinary approach taking into consideration orthognathic surgery can represent a valuable treatment strategy. Both therapies have been shown to improve facial and dental impairments. These improvements extend to related oral functions and overall QoL after treatment [4–7]. Beyond functional rehabilitation, the improvement in facial aesthetics represents a key outcome of both orthodontic treatment (OT) and orthognathic surgical treatment (OST), often influencing patients’ motivation to seek treatment [8–12].

The success of OT and OST is based on achieving functional and stable occlusion in accordance with Andrews’ six keys [13], coordinated arch forms, periodontal and temporomandibular joint health, and harmonious facial proportions [14–17].

According to the World Health Organization (WHO), health is defined as the condition of complete physical, mental, and social well-being rather than merely the absence of disease or infirmity [18]. In this context, Oral Health Related Quality of Life (OHRQoL) reflects patients’ subjective perceptions and can be effectively assessed using validated instruments such as the Oral Health Impact Profile-14 (OHIP-14) and the Orthognathic Quality of Life Questionnaire (OQLQ) [19–21].

The studies by Fleming et al. [22] and Bakes et al. [21] demonstrate an improvement in QoL through orthodontic treatment, with similar benefits observed following OST. Considering the data, the degree of change in OHRQoL may differ for patients in relation to oral sociodemographic distribution and oral health impact profile [16, 23–25].

The aim of this review is to identify the benefits related to OT and OST in terms of QoL improvement, considering the psychosocial aspects of both treatment strategies.

## Materials and methods

### Study design

A systematic review was conducted in adherence with the PRISMA guidelines [26] using the following PICOS framework.

- Population: Adults (≥ 18 years) undergoing orthodontic treatment (OT) or orthognathic surgery (OST).
- Intervention: Orthodontic treatment (fixed appliances or clear aligners).
- Comparison: Orthognathic surgery (traditional or surgery-first approaches).
- Outcomes: OHRQoL, with a focus on psychological impact, assessed using OHIP-14 and OQLQ.
- Study design: RCTs and observational studies (cross-sectional, longitudinal).

It was registered in PROSPERO with number CRD420250655134.

### Search strategy

The following databases were searched in April 2025: PubMed (MEDLINE), EMBASE, Psychinfo, CINAHL and Cochrane Central Register of Controlled Trials (CENTRAL). The search strings used were: “orthodontics” AND “quality of life”; “orthognathic surgery” NOT “congenital abnormalities” AND “quality of life”; the search strings were adjusted to the different search platforms advanced research strategies. Articles published from January 1, 2014 to April, 2025 were searched.

### Study selection

The articles were chosen for analysis according to the following inclusion criteria: only RCTs, prospective and cross-sectional observational studies on adult patients evaluating OHRQoL through the Oral Health Impact Profile Questionnaire (OHIP-14) [27] and/or the Orthognathic Quality of Life Questionnaire (OQLQ) [28], written in English language. Systematic reviews, narrative reviews, case reports, animal and laboratory studies were not considered. Research performed on patients suffering from maxillo-facial trauma, cleft lip and palate, dento-facial syndromic or congenital deformities, and growing patients was discarded, as well as articles using other indices than OHIP-14 and OQLQ. Hand searching was performed by screening the reference lists of included studies and relevant reviews to identify additional eligible articles. Gray literature was not included in searched.

After the identification and removal of duplicates, the retrieved articles underwent a three-step selection protocol: firstly, by title, secondly by abstract and finally full-text.

The study retrieval was independently performed by F.A. and C.M., both specialists in orthodontics with several years of experience in clinical practice and systematic research. The two examiners then proceeded to select the relevant studies for inclusion. Disagreement between reviewers was resolved through discussion, and a third reviewer (N.C.), also a specialist in orthodontics, was consulted if consensus was not reached.

Subsequently, all selected articles underwent data extraction by three independent reviewers (F.A., C.M. and N.C.).

### Quality assessment and Risk of Bias evaluation

The quality of evidence of the selected studies was assessed through the GRADE system (Grading of Recommendations Assessment, Development, and Evaluation) [29]. The risk of bias was evaluated via the Risk of Bias Tool (RoB2) for the randomized controlled trials (RCTs) [30] and via the Risk of Bias in Non-randomized Studies of Exposure Tool (ROBINS-E) for the other studies [31]. Both methods for assessing the risk of bias were developed by the Cochrane Organization.

RoB 2 tool [30] evaluates five main domains: RoB arising from the randomization process, RoB due to deviations from intended interventions, RoB due to missing outcome data, RoB in the measurement of the outcome, RoB in the selection of the reported result.

ROBINS-E tool [31] analyzes seven domains: RoB due to counfounding, RoB arising from measurement of the exposure, RoB in selection of participants, RoB due to post-exposure interventions, RoB due to missing data, RoB arising from measurement of the outcome, RoB in selection of the reported result.

Two independent reviewers (F.A., C.M.) assessed the risk of bias across the domains specified in the chosen tools. Disagreements were resolved through consultation with a third reviewer (N.C.).

### Data extraction

The data extracted from the articles selected for analysis were: year of publication, study design, country in which the study was performed, sample size, patients’ demographics, type of treatment (OT or OST), tool used to assess OHRQoL, timing of OHRQoL evaluation, results (mean and SD).

### Meta-analysis

A meta-analysis was conducted to evaluate the psychological effects of OT compared to OST. The analysis included studies that reported mean and standard deviation values of the OHIP-14 questionnaire as the outcome. For each study, the following parameters were considered: sample size (participants in the questionnaire), mean score and standard deviation for OHIP-14.

For orthognathic surgery, another meta-analysis was performed on studies that reported mean and standard deviation values of the OQLQ questionnaire as the outcome, using the same methodological approach. The same parameters were considered: sample size and mean score and standard deviation for OQLQ.

Analyses were conducted using Prometa3 software (ProMeta 3 – IDoStatistics) [32] and followed a random-effects model, which accounts for two sources of variance: within-study variance and between-studies variance. The overall effect was estimated and graphically represented through a forest plot. Heterogeneity was assessed using I² statistics [33]. A leave-one-out sensitivity analysis was conducted by iteratively removing one study at a time to evaluate the robustness of the pooled effect size and to assess the influence of individual studies on heterogeneity. A p-value < 0.05 was considered statistically significant.

## Results

### Study selection

Using the keywords “orthodontics” AND “Quality of life” 199 studies were identified, while a search for “orthognathic surgery” NOT “congenital abnormalities” AND “quality of life” returned 37 articles. In total, 236 studies were initially retrieved from the databases. After removing duplicates, 189 studies remained. Following the screening of titles and abstracts, 49 articles were selected for further evaluation. After full-text screening, 15 articles were included in this review [34–48].

**Fig. 1:**
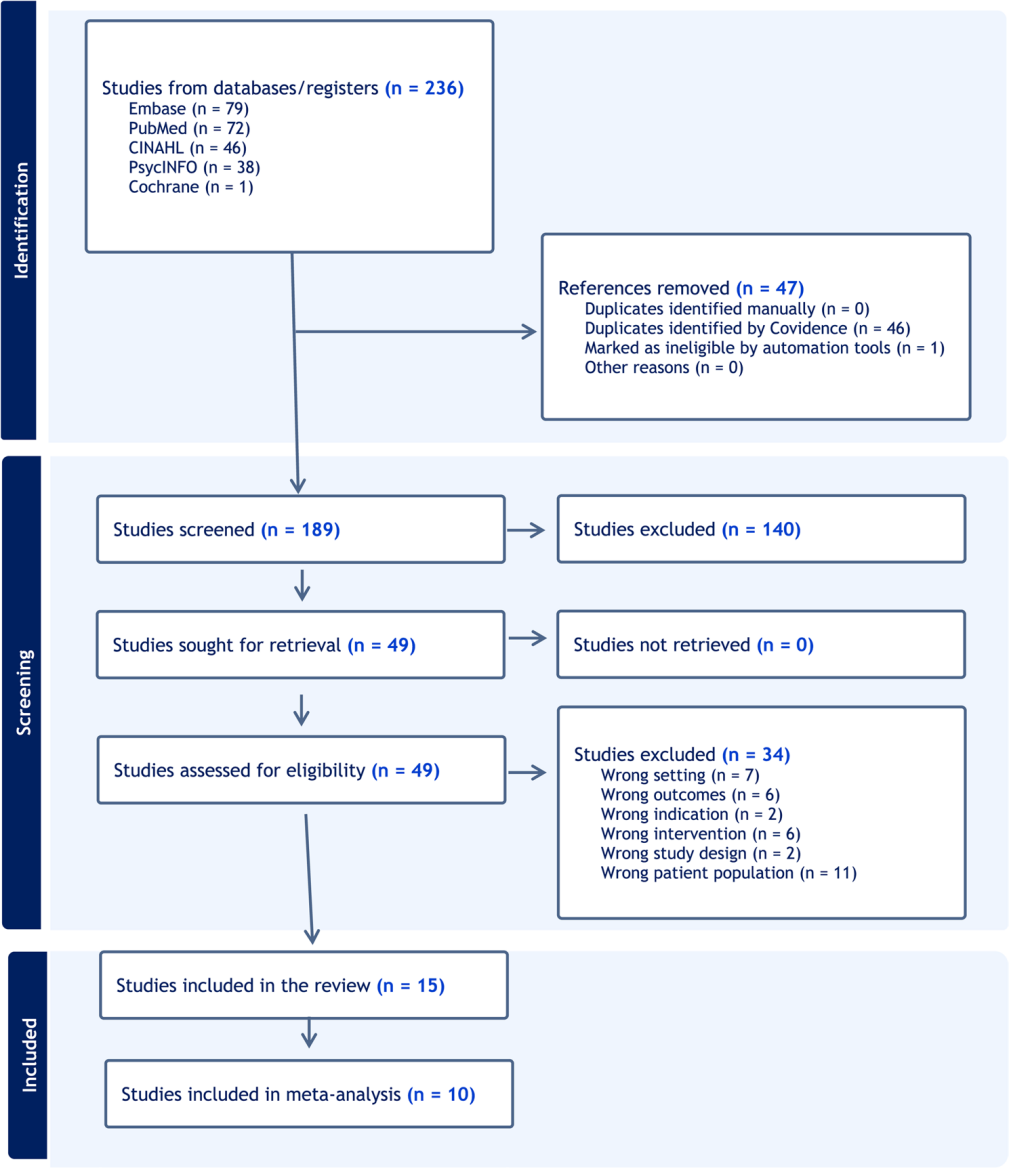
The PRISMA flowchart

The PRISMA flowchart (Fig. 1) illustrates the study selection process, showing the number of studies identified, screened, assessed for eligibility, and included in the review, as well as the reasons for exclusion.

### Studies characteristics

Among the 15 studies included in this review, five were randomized controlled trials (RCTs) [34, 36–38, 45] and ten were observational studies [35, 39–44, 46–48]. Eight studies deal with orthodontics [34–41], while seven studies focused on orthognathic surgery [42–48].

Tables 1 and  2 provide a summary of the main characteristics of orthodontics studies, and orthognathic surgery studies, respectively.

**Table 1:** Characteristics of studies involving orthodontic treatment

Author, year, study design, Country	Sample size	Demographic data	Type of treatment	Tool used to assess OHRQoL	Timing of OHRQoL	Results
Tunca Y, et al. 2024 RCT Turkey	Total (*n* = 60) Group A (= n 30) Group B (= 30)	Age (Mean ± SD): Gr A: 21.3 ± 3.37 Gr B: 23.65 ± 6.58 Gender: Gr A: 15 f – 15 m Gr B: 15 f- 15 m	Orthodontic ◊ Group A (fixed orthodontic) Group B (clear aligners)	OHIP-14 OHRQoL-UK	• The day of device positioning • After 10 days • After 20 days	OHIP-14: *Group A*: • Day 1: 18.73 ± 7.75 • Day 10: 16.50 ± 7.45 • Day 20: 14.53 ± 7.07 *Group B*: • Day 1: 13.07 ± 7.32 • Day 10: 13.10 ± 7.91 • Day 20: 12.43 ± 9.24 OHRQoL-UK questionnaire *Group A*: • Day 1: 45.50 ± 6.16 • Day 10: 45.93 ± 5.29 • Day 20: 47.83 ± 7.65 *Group B*: • Day 1: 46.50 ± 9.34 • Day 48.73 ± 8.02 • Day 20: 49.60 ± 8.87
Pithon MM, et al. 2021 RCT Brazil	Total (*n* = 44) Treated group (*n* = 22) Control group (*n* = 22)	Age (IQR): Treated group: 27.00 (13.00) Control group: 24.00 (15.00) Missing gender data	Orthodontic ◊ -Treated group: space closure -Control group: no orthodontic treatment	OHIP-14	Treated group: • Before orthodontic treatment (baseline) • After space closure (approximately, 3 years after the baseline) Control group: • At the baseline • After 12 months	OHIP-14: *Treated Group*: • Baseline: 31.82 ± 2.82 • Final: 6.91 ± 2.05 *Control group*: • Baseline: 31.50 ± 2.48 • Final: 35.82 ± 1.89
Souza GLN, et al. 2024 Longitudinal study Brazil	Total (*n* = 61) Group 1 (*n* = 33) Group 2 (*n* = 28)	Age (Mean ± SD): Gr 1: 33.73 ± 8.76 Gr 2: 27.11 ± 12.50 Gender: Gr 1: 16 f – 17 m Gr 2: 17 f – 11 m	Orthodontic◊ Group 1 (aligners) Group 2 (fixed appliance)	OHIP-14	During the treatment (not indicated when exactly)	OHIP-14: *Group 1*: 10.21 ± 8.50 *Group 2*: 14.18 ± 8.01
Alfawal AMH, et al. 2022 RCT Syria	Total (= 44) FA group (*n* = 22) CA group (*n* = 22)	Age (Mean ± SD): FA group: 24.22 ± 2.99 CA group: 25.40 ± 2.87 Gender: FA group: 19 f – 3 m CA group: 17 f – 5 m	Orthodontic◊ FA group (fixed appliance) CA group (clear aligners)	OHIP-14	• Before the orthodontic treatment (T0) • 1 week after the treatment initiation (T1) • 1 month after the treatment initiation (T2) • 3 months after the treatment initiation (T3) • 6 months after the treatment initiation (T4) • Post-treatment (T5)	OHIP-14: *FA group*: • T0: 12.5 (11.75–14.25) • T1: 25.18 ± 4.15 • T2: 15.59 ± 2.91 • T3: 11.46 ± 2.63 • T4: 8.59 ± 2.59 • T5: 1 (0-1.25) *CA group*: • T0: 12.5 (10.75-15) • T1: 14.14 ± 3.66 • T2: 9.59 ± 2.70 • T3: 8.18 ± 3.17 • T4: 5.27 ± 2.62 • T5: 0 (0–1)
Choi S.-H, et al. 2017 Longitudinal study Korea	Total (*n* = 66)	Age (Mean ± SD): 24.2 ± 5.2 Gender: 36 f – 30 m	Orthodontic ◊ Fixex appliance	OHIP-14 K	• At baseline (T0) • 12 months after treatment initiation (T1) • At debonding (T2)	OHIP-14: • T0: 10.8 ± 10.2 • T1: 14.1 ± 8.5 • T2: 9.6 ± 7.8
Alhafi ZM, et al. 2024 RCT Syria	Total (*n* = 36) MAA group (*n* = 18) FA group (*n* = 18)	Age (Mean ± SD): MAA gr: 21.89 ± 2.63 FA gr: 20.94 ± 2.38 Gender: MAA gr: 13 f – 5 m FA gr: 12 f – 6 m	Orthodontic ◊ -MAA group (modified aligner appliance with Ni-Ti springs) -FA group (traditional fixed appliance group)	OHIP-14	• Before the treatment (T0) • 2 weeks after the treatment beginning (T1) • 1 month (T2) after the treatment beginning • 2 months (T3) after the treatment beginning • Post-treatment (T4)	OHIP-14: *MAA group*: Mean / Md (Q1–Q3) • T0: 17.56 / 16 (13–21) • T1: 22.72 / 22.5 (18–25) • T2: 14.56/14 (9.75–20.25) • T3: 12.11 / 13 (8.75–14) • T4: 4.83 / 5 (3–6) *FA group*: • T0: 17.61 / 16.5 (15–20) • T1: 19.94 / 19.5 (15–25) • T2: 16.22/14.5 (13–20.25) • T3: 13.6 / 13 (11–14.25) • T4: 4.72 / 5 (4–5)
Al Nazeh AA, et al. 2020 Longitudinal study Saudi Arabia	Total (*n* = 50) Females group (*n* = 26) Males group (*n* = 24)	Age (Mean ± SD): 27.62 ± 8.25 Gender: 26 f – 24 m	Orthodontic ◊ Clear aligners	OHIP-14	• At the baseline, before the treatment • After the treatment	OHIP-14: *Females*: • Before: 14.15 ± 7.052 • After: 8.12 ± 4.803 *Males*: • Before: 10.88 ± 6.873 • After: 12.25 ± 9.275
Kapetanović A, et al. 2022 Cohort study Netherlands	Total (*n* = 42)	Age (Mean ± SD): 27.4 ± 9.3 Gender: 33 f – 9 m	Orthodontic ◊ MARPE	OHIP-14	• At baseline (T0) • In the expansion phase (P1) • Post-expansion phase (P2)	OHIP-14: • T0: 10.86 ± 9.71 • P1: 17.18 ± 10.43 • P2: 9.27 ± 7.92

**Table 2:** Characteristics of studies involving orthognathic treatment

Author, year, study design, Country	Sample size	Demographic data	Type of treatment	Tool used to assess OHRQoL	Timing of OHRQoL	Results
Palomares NB, et al. 2016 Cross- sectional study Brazil	Total (*n* = 254) Group 1 (*n* = 65) Group 2 (*n* = 75) Group 3 (*n* = 62) Group 4 (*n* = 52)	Age (Mean ± SD): Gr 1: 26.6 ± 8.3 Gr 2: 24.8 ± 6.8 Gr 3: 27.9 ± 8.1 Gr 4: 30.1 ± 8.8 Gender: Gr 1: 38 f – 27 m Gr 2: 38 f – 37 m Gr 3: 38 f – 24 m Gr 4: 33 f – 19 m	Orthognathic ◊ Gr 1: Initial (not treated yet) Gr 2: Presurgical Gr 3: Postsurgical Gr 4: Retention	OHIP-14 OQLQ	The interviews were performed before the clinical examinations	OHIP-14 total: *Group 1*: 16.9 ± 12.2 *Group 2*: 13.3 ± 10.5 *Group 3*: 14.4 ± 11.4 *Group 4*: 4.4 ± 4.2 OQLQ total: *Group 1*: 43.5 ± 24.2 *Group 2*: 33.3 ± 23 *Group 3*: 15.4 ± 10.7 *Group 4*: 6.2 ± 4.7
Alanko O, et al. 2017 Longitudinal study Finland	Total (*n* = 44) Surgery patients (test): *n* = 22 Adults not requiring orthognathic treatment (control): *n* = 22	Age (Range / Mean): Surgery patients: 18–54 /36 Control group: 19–49 /25 Gender: Surgery patients: 16 f – 6 m Control group: 22 f	Orthognathic	OQLQ	Surgery patients: • Before the beginning of treatment (T0) • After the first orthodontic examination (T1) • Three times during treatment (T2–T4) • 1 year after surgery (T5) Control group: • At the beginning of the study (T0) • 2 years later (T4) • 4 years after T0 (T5)	OQLQ total (surgery patients): • T0/T1: 31.38 ± 20.71 • T2: 37.67 ± 24.71 • T3: 35.89 ± 23.39 • T4: 35.73 ± 23.58 • T5: 14.50 ± 13.04 Comparison of patiens and control results at T5 (OQLQ total): *Surgery patients*: 14.50 ± 13.04 *Control group*: 21.09 ± 17.27
Bengtsson M, et al. 2018 RCT Sweden	Total (*n* = 62) 3D group (test) (*n* = 31) 2D group (control) (*n* = 31)	Age (Mean): 3D group: 20.5 2D group: 21.1 Gender: 3D group: 15 f – 13 m 2D group: 12 f – 17 m	Orthognathic	OHIP-14	• Before surgery (T0) • 12 months after surgery (T1)	OHIP total: *3D group*: • T0: 53.98 • T1: 27.31 *2D group*: • T0: 61.26 • T1: 23.62
Ni J, et al. 2019 Longitudinal study China	Total (*n* = 45) Patients (*n* = 21) Controls (*n* = 24)	Age (Mean ± SD): Patients: 24.10 ± 3.67 Controls: 24.42 ± 5.31 Gender: Patients: 10 f – 11 m Controls: 15 f – 9 m	Orthognathic ◊ Patients: orthognathic treatment Controls: no treatment	OHIP-14 OQLQ	Patients: • Pre-treatment (T0) • Pre-surgical orthodontic treatment (6 to 8 months, T1) • Post-surgical orthodontic treatment (6 to 8months after surgery, T2)	OHIP total: *Patients*: • T0: 34.2 ± 8.3 • T1: 37.6 ± 9.1 • T2: 18.3 ± 5.2 *Controls*: • T0: 16.5 ± 4.1 • T2: 16.5 ± 4.1 OQLQ total: *Patients*: • T0: 52.4 ± 10.7 • T1: 60.3 ± 11.2 • T2: 28.9 ± 7.5
Feu D, et al. 2016 Longitudinal study Brazil	Total (*n* = 16) OF group (*n* = 8) SF group (*n* = 8)	Missing age and gender data	Orthognathic◊ -OF group (traditional orthodontic-surgical approach) -SF group (surgery first approach)	OHIP-14 OQLQ	• At the baseline (T0) • 1 month after appliance placement (T1) • 3 months after the beginning of the treatment (T2) • 6 months after the beginning of the treatment (T3) • 1 year after the beginning of the treatment (T4) • 2 years after the beginning of the treatment (T5) For SF group there was also an evaluation stage 3–4 weeks after the orthognathic surgery (TPS)	OHIP-14 total: *OF group*: • T0: 21.5 ± 9.0 • T1: 20.4 ± 4.6 • T2: 15.0 ± 6.7 • T3: 11.1 ± 8.7 • T4: 14.1 ± 11.3 • T5: 22.1 ± 11.8 *SF group*: • T0: 25.4 ± 5.6 • T1: 26.9 ± 10.6 • TPS: 20.1 ± 8.0 • T2: 17.0 ± 9.7 • T3: 14.9 ± 11.0 • T4: 7.5 ± 6.6 • T5: 8.1 ± 5.7 OQLQ total: *OF group*: • T0: 17.6 ± 2.5 • T1: 18.8 ± 3.3 • T2: 19.5 ± 3.0 • T3: 17.6 ± 5.6 • T4: 18.5 ± 4.0 • T5: 20.6 ± 1.7 *SF group*: • T0: 21.5 ± 1.1 • T1: 20.4 ± 3.8 • TPS: 12.6 ± 5.3 • T2: 11.5 ± 7.2 • T3: 10.9 ± 6.4 • T4: 6.1 ± 3.6 • T5: 6.4 ± 3.5
Pelo S, et al. 2017 Longitudinal study Italy	Total (*n* = 30) Test group (*n* = 15) Control group(*n* = 15)	Age (Mean ± SD): 30.2 ± 4.3 Gender: 20 f – 10 m	Orthognathic◊ -Test group: surgery-first approach -Control group: conventional orthognathic surgery approach	OHIP-14 OQLQ	• T0: before bracket placement • T1: 1 month preoperatively (for control group) • T2: 1 month postoperatively T	OHIP-14 total: *Test group*: • T0: 16 ± 6 • T2: 2 ± 1 *Control group*: • T0: 13 ± 5 • T1: 18 ± 6 • T2: 3 ± 2 OQLQ total: *Test group*: • T0: 57 ± 10 • T2: 22 ± 3 *Control group*: • T0: 52 ± 10 • T1: 60 ± 9 • T2: 29 ± 9
Paunonen J, et al. 2020 Cohort study Finland	Total (*n* = 123) Group 1: (*n* = 77) Group 2: (*n* = 24) Group 3: (*n* = 22)	Age (Mean ± SD): Gr1: 41 Gr2: 48 ± 9.5 Gr3: 35 ± 13 Gender: Gr1: 71% f – 29% Gr2: 50% f – 50% m Gr3: 86% f – 14% m	Orthognathic (BSSO)◊ -Gr1: already treated -Gr2: already treated (first control group) -Gr3: not treated yet (second control group)	OHIP-14 OQLQ	• Gr1: clinically examined 6 years (range 4–8 years) after BSSO • Gr2: they answered, several years after BSSO, a telephone interview • Gr3: they answered the questionnaire before surgery	OHIP-14 total: *Gr1*: 64.3 ± 8.1 *Gr2*: 64.7 ± 8.4 *Gr23*: 57.6 ± 9.0 OQLQ total: *Gr1*: 12.0 ± 14.0 *Gr2*: 7.5 ± 11.9 *Gr3*: 26.5 ± 17.3

All the articles evaluated OHRQoL either as the primary outcome or as one of several outcomes. Although some studies incorporated additional questionnaires alongside the OHIP and OQLQ (such as the Secord and Jourard Body Image Questionnaire, the Rosenberg Self-Esteem Scale, the Acceptance and Action Questionnaire II, the State-Trait Anxiety Inventory, the Jaw Functional Limitation Scale, the Orofacial Esthetic Scale, the Zung Self-Rating Depression Scale, the Key Subjective Food Intake Ability Questionnaire, the Neuroticism Extraversion Openness Five-Factor Inventory, and the Visual Analogue Scale Questionnaire), only OHIP and OQLQ were considered for the purposes of this review.

### Risk of bias assessment

Overall, the RCTs showed a low risk of bias for most domains, except for “selection of the reported result” where all studies were judged as having “some concerns” (Table 3).

**Table 3:** Table presenting the Risk of Bias assessments for each domain in every RCT study

Study	Randomization RoB	Deviations RoB	Missing data RoB	Measurement RoB	Reporting RoB	Overall Risk of Bias
Tunca Y, et al. (2024)	Some concerns	Low	Low	Some concerns	Some concerns	Some concerns
Bengtsson M, et al. (2018)	Low	Low	Low	Low	Some concerns	Low / Some concerns
Pithon MM, et al. (2021)	Low	Low	Low	Low	Some concerns	Low / Some concerns
Alfawal AMH, et al. (2022)	Low	Low	Low	Low	Some concerns	Low / Some concerns
Alhafi ZM, et al. (2024)	Low	Low	Low	Low	Some concerns	Low / Some concerns

In observational studies, the main sources of bias were “selection of participants” and “selection of the reported result”; furthermore, one of the observational studies showed a “high risk of bias” in the domain of “missing data” (Table 4).

**Table 4:** Table presenting the Risk of Bias assessments for each domain in every observational study

Study	Confouning RoB	Exposure RoB	Seletion RoB	Post-expsure RoB	Missing data RoB	Outcome RoB	Selective reporting RoB	Overall Risk of Bias
Palomares NB, et al. (2016)	Low	Low	Some concerns	Low	Low	Low	Low	Low / Some concerns
Alanko O, et al. (2017)	Some concerns	Low	Low	Low	High	Some concerns	Some concerns	High
Ni J, et al. (2019)	Low	Low/ Some concerns	Some concerns	Low	Low	Low	Some concerns	Some concerns
Souza GLN, et al. (2024)	Low	Some concerns	Low	Low	Some concerns	Some concerns	Some concerns	Some concerns
Choi SH, et al. (2017)	Some concerns	Low	Some concerns	Low	Some concerns	Low	Low	Some concerns
Feu D, et al. (2016)	Some concerns	Low	Some concerns	Low	Low	Some concerns	Some concerns	Some concerns
Pelo S, et al. (2017)	Low	Low	Low	Some concerns	Low	Some concerns	Some concerns	Some concerns
Al Nazeh AA, et al. (2020)	Low	Low	Low	Low	Low	Low	Low	Low
Kapetanović A, et al. (2022)	Some concerns	Low	Some concerns	Low	Low	Low	Low	Some concerns
Paunonen J, et al. (2020)	Low	Low	Some concerns	Low	Some concerns	Low	Low	Some concerns

### Results of individual studies

#### Orthodontics

The reviewed studies on orthodontics (Table 1) collectively indicate that orthodontic treatment has a generally positive effect on OHRQoL, with aligners associated with better outcomes than fixed appliances.

As a matter of fact, in the article by Tunca et al. [34] Group A (fixed appliances) experienced an improvement in OHIP-14 scores over 20 days, with a mean reduction from 18.73 ± 7.75 to 14.53 ± 7.07. In contrast, Group B (clear aligners) showed consistently lower scores, indicating better OHRQoL, with only minimal changes (13.07 ± 7.32 to 12.43 ± 9.24).

Similarly, Souza et al. [35] and Alfawal et al. [36] observed better outcomes in clear aligner groups, the latter reporting near-zero post-treatment scores in that group.

Alhafi et al. [37] found significant improvements for both modified aligner appliances (MAA) and fixed appliances (FA), with MAA achieving slightly better final scores.

In the study by Pithon et al. [38] the treated group (orthodontic space closure with fixed appliances) showed a substantial improvement in OHIP-14 scores, from 31.82 ± 2.82 at baseline to 6.91 ± 2.05 after treatment, while untreated controls worsened (from 31.50 ± 2.48 to 35.82 ± 1.89).

Choi et al. [39] noted an initial decline in OHRQoL during treatment, followed by improvement after debonding.

Al Nazeh et al. [40] found a notable gender-based disparity in outcomes. Females derived greater benefits from clear aligner treatment in terms of improved OHRQoL, whereas males experienced an unexpected increase in reported oral health concerns following treatment. This finding suggests possible differences in compliance, perception, or treatment expectations between genders.

Kapetanović et al. [41], studying MARPE, observed a temporary worsening during expansion followed by improvement post-expansion.

In terms of precision, Alhafi et al. [37], Pithon et al. [38], and Alfawal et al. [36] reported smaller SDs or IQRs, indicating higher precision. In contrast, studies like Tunca et al. [34], Souza et al. [35], and Kapetanović et al. [41] had higher variability, particularly during treatment phases. Al Nazeh et al. [40] also showed more precise outcomes in females, while males exhibited greater variability.

#### Orthognathic surgery

Studies on orthognathic surgery (Table 2) show significant improvements in OHRQoL, with outcomes varying by timing, treatment approach, and assessment tools.

Orthognathic surgery seems to improve multiple OHRQoL dimensions, including self-esteem, social interaction, and oral function. These benefits are most evident in the retention phase, as seen in Palomares et al. [42], where the retention group had the lowest OHIP-14 (4.4 ± 4.2) and OQLQ (6.2 ± 4.7) scores.

Long-term benefits were confirmed by Alanko et al. [43] and Paunonen et al. [44], with treated patients maintaining significantly better OHRQoL compared to untreated controls.

For instance, Alanko et al. reported post-treatment OQLQ = 14.50 ± 13.04 in the surgery group vs. 21.09 ± 17.27 in controls; Paunonen et al. found better outcomes in treated patients (Group 1: OQLQ = 12.0 ± 14.0) than in controls (Group 3: OQLQ = 26.5 ± 17.3).

Bengtsson et al. [45] showed improvements in both 3D and 2D groups, with slightly better OHIP scores in the 2D group (T1 = 23.62 vs. 27.31). Ni et al. [46] also found significant post-surgical improvements (OHIP = 18.3 ± 5.2; OQLQ = 28.9 ± 7.5), while control scores remained stable.

The surgery-first approach (SF) was shown to be particularly effective in achieving faster and more sustained OHRQoL gains. Feu et al. [47] reported post-treatment scores of OHIP = 8.1 ± 5.7 and OQLQ = 6.4 ± 3.5 in the SF group. Similarly, Pelo et al. [48] found superior outcomes in the SF group (T2: OHIP = 2 ± 1; OQLQ = 22 ± 3) compared to controls.

Regarding precision, Ni et al. [46] and Pelo et al. [48] showed narrower SDs, suggesting more reliable effect estimates, while most other studies presented higher variability and thus lower precision.

### Data synthesis

This review assessed OHRQoL at various stages of OT and OST using OHIP-14 and OQLQ questionnaires. Significant post-treatment improvements were observed, particularly after surgery or orthodontic debonding. Faster or less invasive treatments (e.g., clear aligners, surgery-first approaches) often led to better OHRQoL outcomes.

Qualitative assessment revealed substantial variability among the included studies. This variability is related to differences in patient characteristics (e.g., age, gender, malocclusion severity, general health), as well as differences in sample sizes and study designs (RCTs vs. observational).

Additional variability arose from questionnaire factors: language versions, administration methods (electronic vs. paper) and timing of data collection (pre/post-treatment or long-term follow-up).

Moreover, OQLQ questionnaire evaluates both aesthetic and functional aspects, while OHIP questionnaire is more focused on functional aspects and general oral well-being.

It should also be considered that some studies focused on a single treatment modality, while others compared different therapeutic approaches. Differences in follow-up duration and the stability of outcomes further complicated comparisons.

### Certainty of evidence

The quality of evidence for all studies included in the review was evaluated using the GRADE system [29]. Table 5 provides an assessment of each study across five key domains: Risk of Bias, Inconsistency, Indirectness, Imprecision, and Publication Bias, along with the overall GRADE Certainty rating.

**Table 5:** Quality of evidence of all studies according to the GRADE system

Study	RoB	Inconsistency	Indirectness	Imprecision	Publication Bias	GRADE Certainty
Tunca Y, et al. 2024	Moderate	Moderate	Low	Moderate	Moderate	Moderate
Pithon MM, et al. 2021	Low	Low	Low	Low	Low	High
Souza GLN, et al. 2024	Moderate	Low	Low	Moderate	Moderate	Moderate
Alfawal AMH, et al. 2022	Low	Low	Low	Low	Low	High
Choi S.-H, et al. 2017	High	Low	Low	High	High	Low
Alhafi ZM, et al. 2024	Low	Low	Low	Moderate	Low	Moderate
Al Nazeh AA, et al. 2020	Moderate	Low	Low	Moderate	Moderate	Moderate
Kapetanović A, et al. 2022	Low	Low	Low	Moderate	Low	Moderate
Palomares NB, et al. 2016	Low	Low	Low	Low	Low	High
Alanko O, et al. 2017	Moderate	Moderate	Moderate	High	Moderate	Low
Bengtsson M, et al. 2018	Moderate	Low	Low	Moderate	Moderate	Moderate
Ni J, et al. 2019	Moderate	Moderate	Low	Moderate	Moderate	Moderate
Feu D, et al. 2016	Moderate	Moderate	Low	Moderate	Moderate	Moderate
Pelo S, et al. 2017	Low	Low	Low	Low	Low	High
Paunonen J, et al. 2020	Moderate	Moderate	Low	Moderate	Moderate	Moderate

### Meta-analysis

The meta-analysis conducted to compare OT and OST included 10 studies that assessed psychological outcomes using the OHIP-14 questionnaire after treatment. Since different studies evaluated patients at varying time points, the last available assessment from each study was considered, even though these time points were not always consistent.

Several studies were excluded from the meta-analysis for specific reasons: Souza et al. [35] was not included because the questionnaire was administered during the treatment period. Alhafi et al. [37] was excluded as it reported OHIP-14 results using mean, median, and quartiles but did not provide standard deviation values. Alanko et al. [43] was omitted because it only reported results for the OQLQ questionnaire. Bengtsson et al. [45] was also excluded due to the absence of standard deviation data. Additionally, Paunonen et al. [44] administered the questionnaire six years post-treatment, a significantly longer follow-up period compared to the other studies, making it unsuitable for inclusion.

Two studies in the orthodontic group (Alfawal et al., 2022, and Tunca et al., 2024) and two in the surgery group (Feu et al., 2016, and Pelo et al., 2017) evaluated treatment outcomes by comparing two subgroups: clear aligners vs. fixed appliances in the orthodontic group and the traditional approach vs. surgery-first approach in the surgical group. To prevent data loss and ensure their inclusion in the meta-analysis, each subgroup was treated as a separate study, resulting in double entries in the final analysis.

**Fig. 2:**
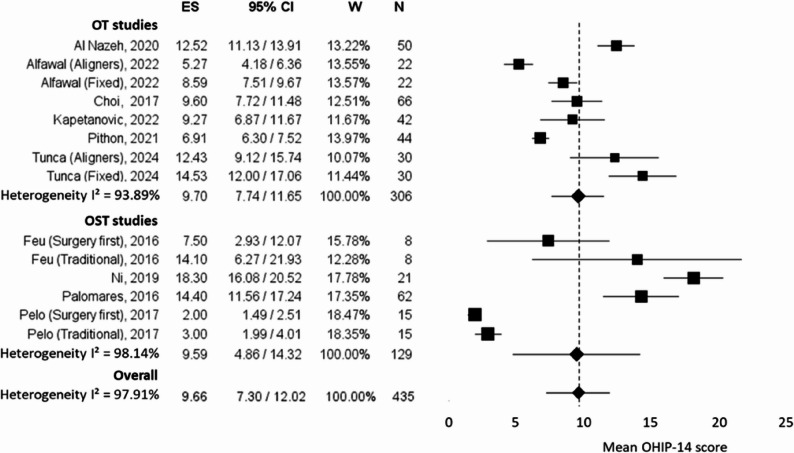
Forest plot reporting the mean score obtained from OHIP-14 questionnaire from orthodontic (OT) and orthognathic (OST) studies, along with their 95% confidence intervals (CIs). The x-axis represents the mean OHIP-14 score (effect size) reported in each study, with higher values indicating worse oral health–related quality of life. Each square represents the mean effect estimate for an individual study, with the size of the square proportional to the study’s weight in the meta-analysis. The horizontal lines indicate the 95% CIs. The diamond at the bottom of each subgroup represents the pooled estimate for orthodontic and orthognathic studies, respectively. The overall pooled effect estimate from the random-effects model is shown at the bottom of the plot. The vertical dotted line represents the overall pooled mean effect estimated using the random-effects model. Studies plotted to the left of this line reported lower mean OHIP-14 scores than the pooled average, whereas studies plotted to the right reported higher mean scores. Heterogeneity is quantified using the I² statistic. *ES* Effect size, *CI* Confidence intervals, *W* Weights, *N* Sample size

As a result, the metanalysis included 14 studies with a total sample of 435 patients who underwent either orthodontic treatment (*n* = 306) or orthognathic surgery (*n* = 129). The overall effect size was 9.66 (95% CI: 7.30–12.02), indicating the mean psychological impact of both treatments. As shown in the forest plot in Fig. 2 the subgroup analysis revealed a similar effect size for both orthodontic (9.70, 95% CI: 7.74–11.65) and orthognathic surgery (9.59, 95% CI: 4.86–14.32) groups, with statistically significant results (*p* < 0.01 for both).

**Fig. 3:**
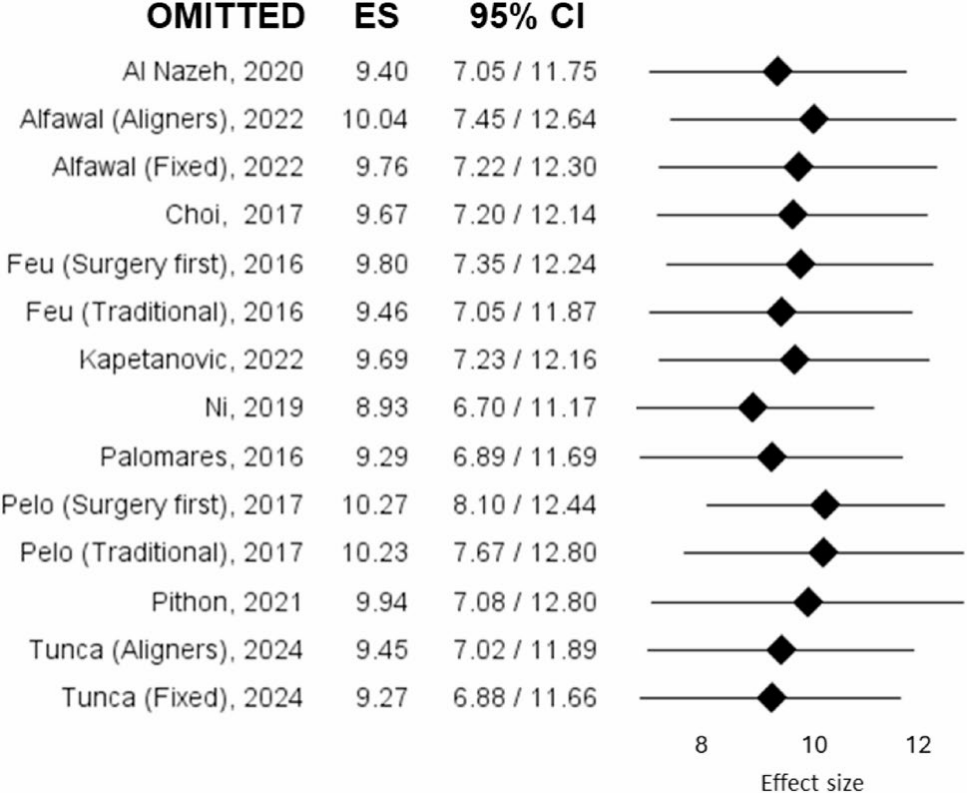
Leave-one-out sensitivity analysis of the meta-analysis on OHIP-14 scores. Each row represents the pooled effect size recalculated after sequentially excluding one study. Squares indicate the re-estimated pooled mean, and horizontal lines represent 95% confidence intervals. The analysis shows the influence of individual studies on the overall effect size. *ES* Effect size, *CI* Confidence intervals

Heterogeneity was high across studies, with an I² of 97.91% overall, suggesting considerable variability in the included studies. The leave-one-out sensitivity analysis demonstrated that exclusion of any individual study did not substantially influence the pooled effect size (range: 8.93–10.27) or the level of heterogeneity (I² range: 96.13%–98.07%). These findings suggest that the high heterogeneity observed in the overall meta-analysis was not driven by a single study. The forest plot of the leave-one-out analysis for the OHIP-14 meta-analysis is presented in Fig. 3, while detailed heterogeneity estimates are reported in Supplementary Table 1.

The orthodontic group exhibited an I² of 93.89%, while the surgery group showed even greater heterogeneity (I² = 98.14%), which may be attributed to differences in study designs, patient populations, and assessment instants. The variance was notably higher in the surgery group (5.83) compared to the orthodontic group (0.99), reflecting greater variability in psychological outcomes among surgical patients.

The results of the ANOVA Q-test indicated that there was no statistically significant difference between the orthodontic and surgical groups in terms of psychological effects post-treatment (Q = 0.0018, *p* = 0.966). This suggests that both treatments had a comparable psychological impact on patients, with variability within studies but no clear distinction between the two approaches. However, the high overall heterogeneity indicated that other factors (such as patient expectations, recovery duration, and psychological support) might influence the subjective post-treatment experience and warrant further investigation.

**Fig. 4:**
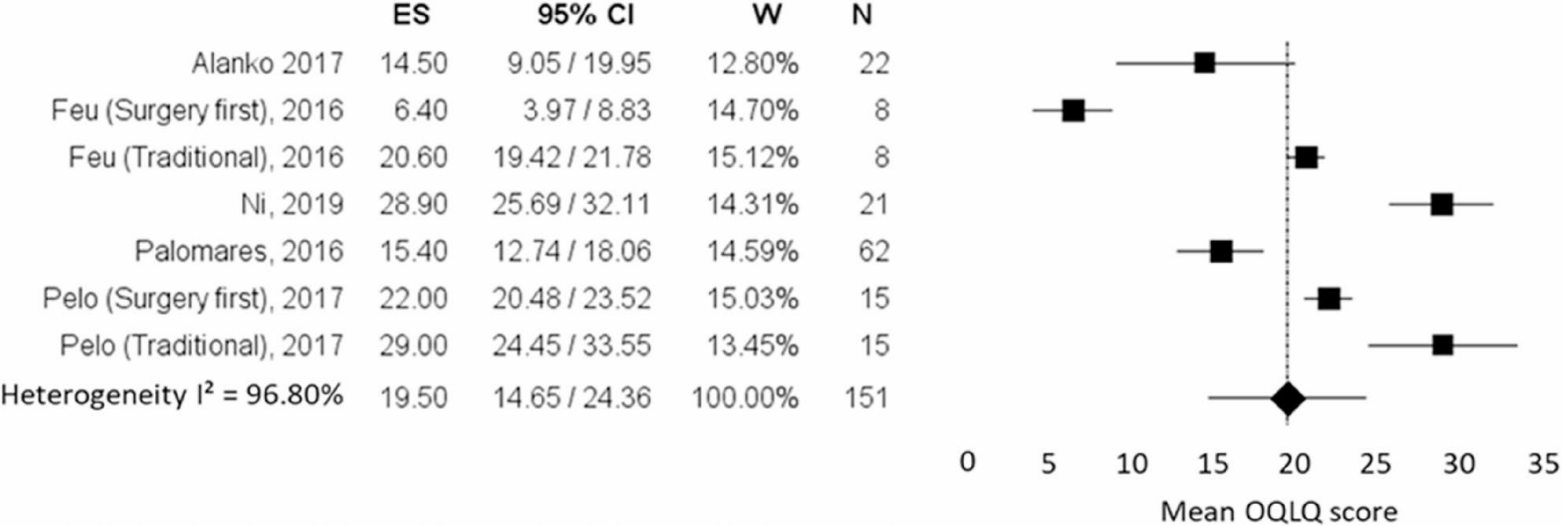
Forest plot summarizing the mean OQLQ scores from orthognathic surgery (OST) studies, along with their 95% confidence intervals (CIs). The x-axis represents the mean OQLQ score (effect size) reported in each study, with higher values indicating worse oral health–related quality of life. Each square represents the mean effect estimate for an individual study, with the size of the square proportional to the study’s weight in the meta-analysis. The horizontal lines indicate the 95% CIs. The overall pooled effect estimate from the random-effects model is shown at the bottom of the plot. The vertical dotted line represents the overall pooled mean effect estimated using the random-effects model. Studies plotted to the left of this line reported lower mean OQLQ scores than the pooled average, whereas studies plotted to the right reported higher mean scores. Heterogeneity is quantified using the I² statistic. *ES* Effect size, *CI* Confidence intervals, *W* Weights, *N* Sample size

**Fig. 5:**
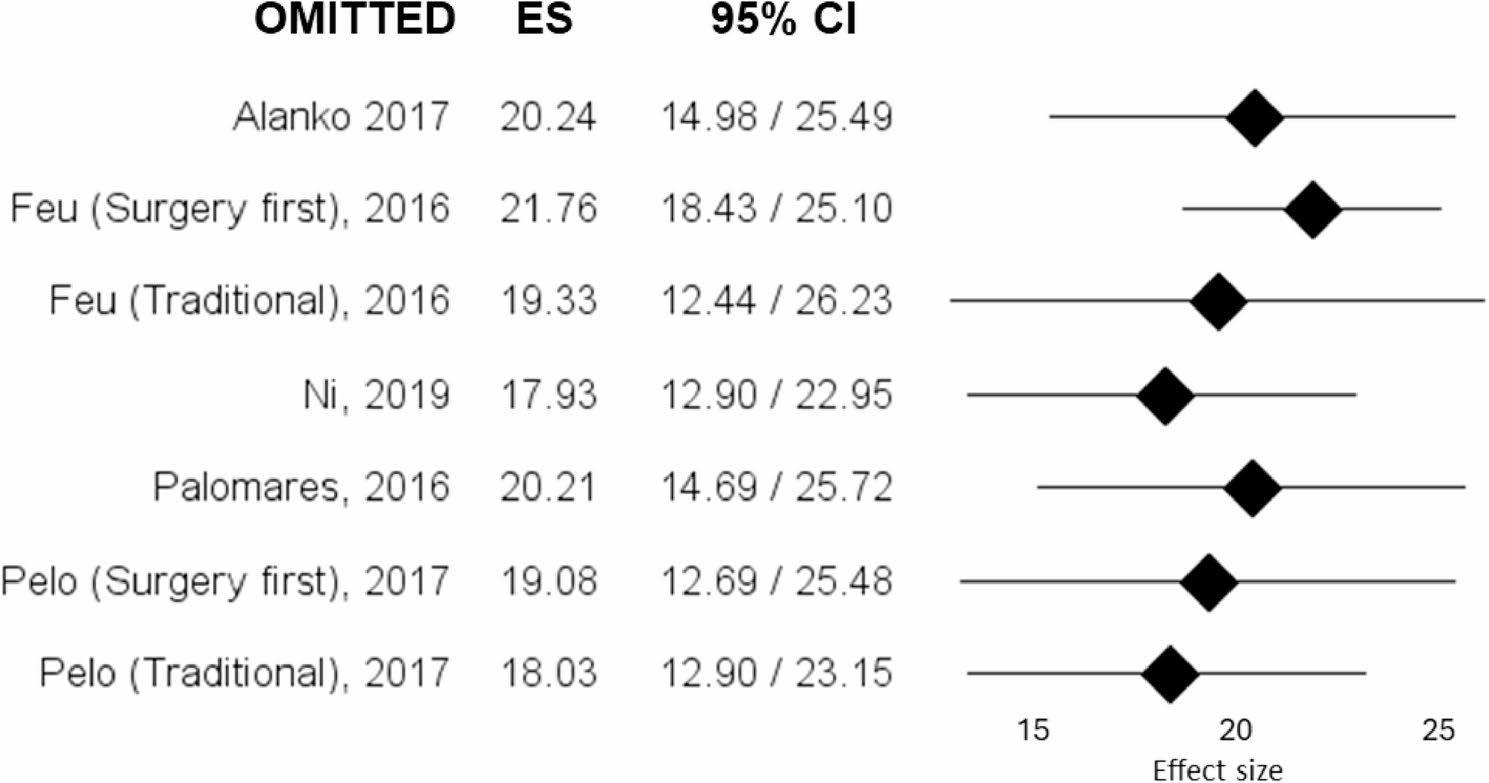
Leave-one-out sensitivity analysis of the meta-analysis on OQLQ scores. Each row represents the pooled effect size recalculated after sequentially excluding one study. Squares indicate the re-estimated pooled mean, and horizontal lines represent 95% confidence intervals. The analysis shows the influence of individual studies on the overall effect size. *ES* Effect size, *CI* Confidence intervals

For the meta-analysis conducted on orthognathic studies, 6 studies reporting mean and standard deviation values of OQLQ were included: Alanko et al. [43], Ni et al. [46], Palomares et al. [42]; Pelo et al. [48] and Feu et al. [47] were considered as double entries (Traditional and Surgery-first approach). As a result, the metanalysis included 7 studies with a total sample of 151 patients. The overall effect size was 19.50 (95% CI: 14.65–24.36), and I² = 96.80%, as represented in Fig. 4. The variance was notably high (6.13), reflecting great variability in psychological outcomes among surgical patients also with this questionnaire. As for the previous meta-analysis, the leave-one-out sensitivity analysis demonstrated that exclusion of any individual study did not substantially influence the pooled effect size (range: 17.93–21.76) or the level of heterogeneity (I² range: 91.60%–97.29%). The forest plot of the leave-one-out analysis for the OQLQ meta-analysis is presented in Fig. 5, while detailed heterogeneity estimates are reported in Supplementary Table 2.

## Discussion

Evaluating the psychological effects of OT versus OST is the key to understanding their broader impacts.

Orthognathic surgery, aimed at achieving both functional and aesthetic improvements, consistently improves OHRQoL. Nevertheless, the psychological effects of OST fluctuate across different treatment phases.

Studies by Palomares et al. [42] and Alanko et al. [43] highlighted a relatively high psychological burden in the pre-surgical phase, reflected by elevated OHIP-14 and OQLQ scores. Specifically, Palomares et al. found that pre-surgical patients (Group 2) had OQLQ scores of 33.3 ± 23, higher than post-surgical patients, indicating heightened distress.

Alanko et al. reported similar trends, with OQLQ scores increasing during pre-surgical phase (T2–T4) before detecting a notable improvement post-surgery (T5: 14.50 ± 13.04).

On the contrary, post-surgical phase registered a substantial improvement: Ni et al. [46] demonstrated a dramatic decrease in OQLQ scores post-surgery (T2: 28.9 ± 7.5, compared to pre-treatment T0: 52.4 ± 10.7). Similarly, Bengtsson et al. [44] observed OHIP reductions one year after surgery (3D group: T0: 53.98 to T1: 27.31).

Regarding the retention and long-term effects Paunonen et al. [44] found long-term stability in psychological improvements after bilateral sagittal split osteotomy (BSSO), with treated patients (Group 1) showing significantly better OQLQ scores (12.0 ± 14.0) compared to untreated controls (26.5 ± 17.3).

Considering surgical approaches and timing, Pelo et al. [48] compared conventional and surgery-first approaches, finding that the surgery-first group reported faster psychological relief, with OHIP scores dropping to 2 ± 1 within one month postoperatively (T2).

Feu et al. [47] further reported that surgery-first approaches yielded quicker OHRQoL improvements than traditional methods.

On the other hand, orthodontic treatment shows more gradual QoL improvements compared to the immediate changes observed with orthognathic surgery. Overall, the psychological impact tends to be less profound.

These findings are consistent with previous evidence showing that orthodontic treatment leads to significant improvements in OHRQoL over time, despite an initial period of discomfort and functional limitations, as reported by Jamilian et al. [49].

Furthermore, initial phases of OT can cause temporary discomfort and challenges in daily functioning, particularly in terms of pain and chewing difficulties, as evidenced by Choi et al. [39], where OHIP-14 K scores increased during the first year of treatment (T0: 10.8 ± 10.2 to T1: 14.1 ± 8.5).

Similarly, Tunca et al. [34] found that both Group A and Group B had higher OHIP scores on day 1 (Group A: 18.73 ± 7.75; Group B:13.07 ± 7.32) compared to day 20 (Group A: 14.53 ± 7.07; Group B: 12.43 ± 9.24). As a result, OT may have negative effects on OHRQoL, as supported by some previous studies [50, 51]. However, these negative effects, in most situations, are temporary.

Psychological improvements tend to become more consistent as the OT advances with a marked reduction in discomfort and a positive shift in patients perceived QoL. This trend was observed in in Pithon et al. [38], where OHIP scores dropped from 31.82 ± 2.82 at baseline to 6.91 ± 2.05 post-treatment, emphasizing progressive stabilization of psychological well-being. As well in the article by Alhafi ZM et al. [37] both the MAA and FA groups revealed gradual psychological improvements. For the MAA group, OHIP-14 scores decreased from T1 (22.72 / 22.5) to T4 (4.83 / 5). Similarly, the FA group saw a progressive reduction in distress from T1 (19.94 / 19.5) to T4 (4.72 / 5), indicating consistent improvements over time.

Considering the substantial heterogeneity observed across studies, with an overall I² of 97.91%, caution should be taken in drawing conclusions from the meta-analysis. As assessed using the OHIP-14 questionnaire, the pooled effect size of 9.66 (95% CI: 7.30–12.02) suggests a significant overall impact on patients’ psychological well-being following both OT and OST treatment. Notably, the subgroup analysis revealed similar effect sizes between the orthodontic (9.70, 95% CI: 7.74–11.65) and orthognathic surgery (9.59, 95% CI: 4.86–14.32) groups, indicating comparable improvements in patient-reported quality of life regardless of the treatment modality.

However, due to the high heterogeneity and different psychological trajectories of OT and OST, these pooled results should be interpreted cautiously.

An additional source of heterogeneity may be the lack of stratification by malocclusion type, as most studies included mixed skeletal classes without class-specific psychological outcomes, limiting class-based interpretations.

The results of the ANOVA Q-test further support the conclusion that there was no statistically significant difference between the two treatment groups in terms of psychological impact. The orthodontic group exhibited an I² of 93.89%, while the surgery group showed even greater heterogeneity (I² = 98.14%). This variability may stem from differences in study designs, sample characteristics, follow-up periods, and variations in psychological adaptation to treatment. The notably higher variance in the surgery group (5.83) compared to the orthodontic group (0.99) suggests greater variability in psychological responses among surgical patients, potentially influenced by factors such as the invasiveness of the procedure and recovery duration.

The secondary meta-analysis focusing on the OQLQ questionnaire obtained similar results in terms of heterogeneity and variance. The pooled effect size was 19.50 (95% CI: 14.65–24.36), with an I² of 96.80%. The variance was high (6.13), further highlighting the considerable variability in psychological outcomes among surgical patients assessed using this alternative questionnaire.

The identified biases suggest caution when interpreting the results for observational studies. For both meta-analyses, the leave-one-out sensitivity analysis confirmed the robustness of the present findings, as exclusion of any individual study did not result in a meaningful change in the pooled effect size nor in a substantial reduction of heterogeneity. Heterogeneity remained consistently high across all iterations, indicating that between-study variability was not driven by a single influential study but rather reflects inherent clinical and methodological differences among the included studies. These differences likely include variability in treatment modality (orthodontic treatment versus orthognathic surgery), treatment protocols (surgery-first versus conventional approaches), appliance type (aligners versus fixed appliances), follow-up duration, baseline severity of malocclusion, and population characteristics. Therefore, the observed heterogeneity should be interpreted as a reflection of the diversity of clinical scenarios represented in the current literature rather than as a methodological limitation attributable to outlier effects.

However, the more robust methodologies of the included RCTs strengthen the overall conclusions of this review.

Overall, these findings emphasize the importance of adopting a standardized approach regarding sample size, study design, and assessment time points in studies using the OQLQ and OHIP-14 questionnaires.

In the pre-treatment period it is essential to investigate patient expectations in both treatment approaches, since the psychological impacts of both therapies can be influenced by the patient’s personal initial goals.

The psychological effects of OT can be also influenced by duration of treatment, with longer treatment periods often correlating with more pronounced psychological distress.

OST tends to have a more immediate and dramatic psychological impact, with patients experiencing both high levels of preoperative anxiety and post-operative satisfaction mainly due to enhancing self-esteem and social interactions linked to facial aesthetics improvement. An important aspect in OST is that the psychological impact post treatment is influenced by the information provided by practitioners prior to surgery, as discussed by Zamboni et al. [52]. Properly informed patients tend to be more accepting of treatment and more compliant with therapy, resulting in improved overall outcome in terms of healing, psychological impact, and social life.

Therefore, it is crucial to provide thorough information about all aspects of the therapeutic process before the treatment. Adequate pre-treatment education ensures a clear understanding of the procedure and the potential outcomes aligning expectations with the actual treatment experience and enhancing patient satisfaction, as described in previous articles [53, 54].

Our findings are based on studies conducted in several European countries, which may support the generalizability of the results within this context. However, cultural differences and variations in healthcare systems across countries could influence patient-reported outcomes, including perceptions of treatment-related discomfort and psychological benefits. Therefore, caution is advised when extrapolating these results to non-European populations.

Additionally, while the OHIP questionnaire is a widely used tool to assess OHRQoL, other approaches, such as qualitative studies using interviews, may provide complementary insights into patients’ experiences and perspectives. Incorporating multiple methods to evaluate patient-reported outcomes could enhance understanding of both the quantitative and subjective aspects of treatment impact.

### Limitations

This review provides valuable insights into psychological effects of OST and OT, though several limitations exist, including variations in samples size, questionnaire administration methods, data collection, study designs. Additionally, some studies compared different treatments, while others focused on a single approach. A further limitation is the heterogeneity in assessment time points across the included studies, which complicates direct comparisons of psychological outcomes and may contribute to the high levels of statistical heterogeneity observed in the meta-analysis. Another important limitation is the lack of stratification by malocclusion type (e.g., skeletal Class II vs. Class III), as the majority of studies included heterogeneous samples without class-specific psychological data, potentially contributing to the high heterogeneity and limiting class-specific conclusions. 

Accordingly, the pooled meta-analysis of OT and OST should be considered exploratory, as high heterogeneity limits direct comparability. Future studies with standardized assessment timing and separate analyses are needed.

## Conclusions

Both orthognathic surgery and orthodontic treatment tend to improve OHRQoL, though in distinct mechanisms and timelines.

Psychologically, OST has a more profound impact, but involves a longer recovery and initial emotional distress due to its invasiveness. OT, while generally associated with more moderate psychological changes, offers a less invasive approach, with aligners associated with better OHRQoL outcomes than fixed appliances.

The timing of psychological improvements also differs: OT, whether with fixed appliances or clear aligners, promotes a gradual enhancement in OHRQoL over time. Conversely, OST often leads to more immediate improvements, particularly in psychosocial sphere, with the most notable benefits emerging in the post-surgical phase. The surgery-first approach offers earlier psychological relief compared to traditional protocols.

Although both treatments improve OHRQoL, this review shows no statistically significant difference between them in terms of psychological impact after treatment.

Future studies should focus on long-term psychological effects and the role of patient education and support in both treatments to better manage psychological well-being throughout the process.

## Data Availability

All data generated or analyzed during this study are included in this published article. Additional details are available from the corresponding author upon reasonable request.

## Abbreviations

OT: Orthodontic Treatment
OST: Orthognathic Surgery
OHRQoL: Oral Health-Related Quality of Life
OHIP-14: Oral Health Impact Profile – 14
OQLQ: Orthognathic Quality of Life Questionnaire
RCT: Randomized Controlled Trial
PRISMA: Preferred Reporting Items for Systematic Reviews and Meta-Analyses
GRADE: Grading of Recommendations Assessment, Development and Evaluation
RoB2: Risk of Bias 2 Tool
ROBINS-E: Risk Of Bias In Non-randomized Studies – of Exposures
